# Acute stress enhances the expression of neuroprotection- and neurogenesis-associated genes in the hippocampus of a mouse restraint model

**DOI:** 10.18632/oncotarget.7225

**Published:** 2016-02-06

**Authors:** Giuseppina Sannino, Lorenza Pasqualini, Eugenia Ricciardelli, Patricia Montilla, Laura Soverchia, Barbara Ruggeri, Silvia Falcinelli, Alessandra Renzi, Colleen Ludka, Thomas Kirchner, Thomas G. P. Grünewald, Roberto Ciccocioppo, Massimo Ubaldi, Gary Hardiman

**Affiliations:** ^1^ Department of Medicine, School of Medicine, University of California, La Jolla, California, USA; ^2^ Facoltà di Scienze, Università Politecnica delle Marche, Ancona, Italy; ^3^ Laboratory for Pediatric Sarcoma Biology, Institute of Pathology of the LMU Munich, Munich, Germany; ^4^ Department of Urology, Medical University of Innsbruck, Innsbruck, Austria; ^5^ Centre de Recherches en Cancérologie de Toulouse – CRCT, Toulouse, France; ^6^ School of Pharmacy, Pharmacology Unit, University of Camerino, Camerino, Italy; ^7^ Medical Research Council – Social, Genetic and Developmental Psychiatry Centre, Institute of Psychiatry, King's College London, London, United Kingdom; ^8^ Institute of Pathology of the LMU Munich, Munich, Germany; ^9^ Departments of Medicine, Public Health and Center for Genomics Medicine, Medical University of South Carolina, Charleston, South Carolina, USA

**Keywords:** acute, restraint stress, hippocampus, neurogenesis, neuroprotection

## Abstract

Stress arises from an external demand placed on an organism that triggers physiological, cognitive and behavioural responses in order to cope with that request. It is thus an adaptive response useful for the survival of an organism. The objective of this study was to identify and characterize global changes in gene expression in the hippocampus in response to acute stress stimuli, by employing a mouse model of short-term restraint stress. In our experimental design mice were subjected to a one time exposure of restraint stress and the regulation of gene expression in the hippocampus was examined 3, 12 and 24 hours thereafter. Microarray analysis revealed that mice which had undergone acute restraint stress differed from non-stressed controls in global hippocampal transcriptional responses. An up-regulation of transcripts contributing directly or indirectly to neurogenesis and neuronal protection including, *Ttr*, *Rab6*, *Gh*, *Prl*, *Ndufb9* and *Ndufa6*, was observed. Systems level analyses revealed a significant enrichment for neurogenesis, neuron morphogenesis- and cognitive functions-related biological process terms and pathways. This work further supports the hypothesis that acute stress mediates a positive action on the hippocampus favouring the formation and the preservation of neurons, which will be discussed in the context of current data from the literature.

## INTRODUCTION

Stress is an adaptive response to external demands. In contrast with the well documented negative effects of long-term stress, normal or acute stress provides many beneficial advantages. The release of stress mediators have a protective function for brief periods and prepare an organism to cope with the external requests. Previous studies have demonstrated that acute stress facilitates the ‘fight or flight’ response promoting survival actions, and improving memory and immune system responses [1]. Stress leads to prosocial action in immediate need situations, with adaptive and altruistic effects under conditions that promote survival and well-being at both the individual and group level [2]. Stressful aversive events are well remembered and provide survival benefits. However, maladaptation in stress responses can result in mental illness, such as posttraumatic stress disorder (PTSD) [3].

Stress profoundly affects brain structures, such as the hippocampus, and important physiological processes are mediated by this area [4]. The hippocampus, as part of the limbic system, plays a role in learning, spatial memory, navigation and it is implicated in the pathophysiology of mood disorders [5-8]. Loss of hippocampal neurons due to exposure to stress has been reported in preclinical [9-11] and clinical studies, such as PTSD, borderline personality disorder and major depressive disorder [12-14].

To date, transcriptional profiling of hippocampus has been performed mainly in relation to neurodegenerative and neuropsychiatric disorders, including Alzheimer's disease (AD) and schizophrenia, or studies of neural function on the context of pathologies, such as diabetes [15-17]. The impact of stress on hippocampal gene expression has been previously investigated by research groups including ours utilizing microarrays, but, primarily in association with chronic stress outcomes [18-20]. In this work, a mouse model of short-term restraint stress was adopted to identify changes in gene expression in the hippocampus in response to acute stress. Animals were exposed to a restraint stressor treatment, while control animals, not subjected to the stressor, provided a measure of basal gene expression levels. Genome wide expression profiling data of the hippocampus was obtained 3, 12 and 24 hours following treatment to allow longitudinal changes in expression to be monitored.

Our findings demonstrated that mice exposed to acute restraint stress differed in gene expression patterns compared to the control group. In particular, we noted an up regulation of genes involved in neurogenesis, neuronal protection and defense against oxidative stress.

## TRANSCRIPTIONAL PROFILING OF HIPPOCAMPUS IN RESPONSE TO ACUTE RESTRAINT STRESS BY GENOME WIDE EXPRESSION ANALYSIS

**Figure 1:**
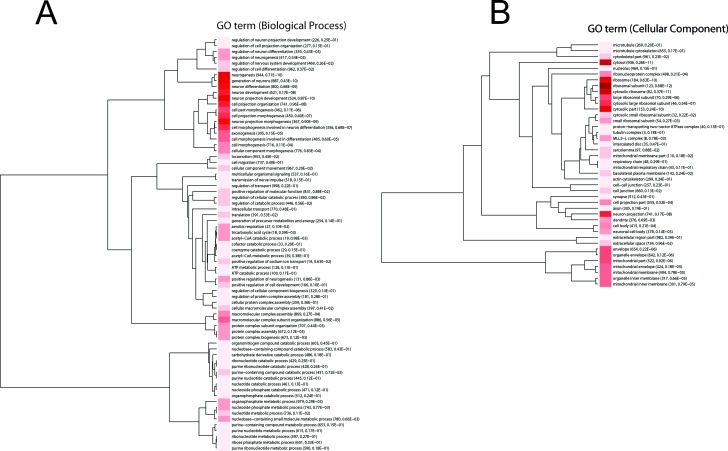
Information clustering of significant (Bonferroni adjusted *p*-value < 0.01) biological process **A.** and cellular component terms **B.** in the hippocampus from the comparison of control and mice exposed to acute stress at 3 h, 12 h and 24 h. Animal were treated as described in [20]. The stress to which the mice were subjected is ‘restraint stress’ where mice were immobilized in restraint tubes for 60 minutes (min) to induce a one-time stress response. Numbers in parentheses are the number of expressed genes in the process and adjusted *p*-value. Hue of the red color is proportional to (log) *p-value*; darker color represents higher statistical significance.

In order to assess the effects of acute stress on hippocampal gene expression pattern, mice exposed to acute restraint stress (27 stressed and 9 control mice) were subjected to whole transcriptome expression analysis. Specifically, changes in gene expression were investigated at 3, 12 and 24 hours (h) following exposure to restraint stress. The data analysis approach followed the logic that transcripts, whose expression patterns were altered at any point across the time course, were of interest from a biological perspective. We ranked these transcripts using a method we have described previously for analyzing time course data [21]. A systems level analysis was then carried out on the entire ranked probe set and significant gene sets were subjected to Gene Ontology (GO) analysis and clustered using variation of information (*VI*) as the distance metric [22-24] (Figure 1A-1B). Only gene sets with adjusted *p*-values ≤ 0.01 were included in these analyses.

Significant enriched GO biological process terms in the mice exposed to acute stress included ‘neurogenesis’, ‘generation of neurons’, ‘neuron differentiation’, ‘neuron projection development and morphogenesis’ (Figure 1A). Significant enriched GO cellular component terms included ribonucleoprotein complex, ribosome, ribosomal subunit, cytosolic ribosome, large ribosomal subunit, cytosolic large ribosomal subunit (Figure 1B).

**Figure 2:**
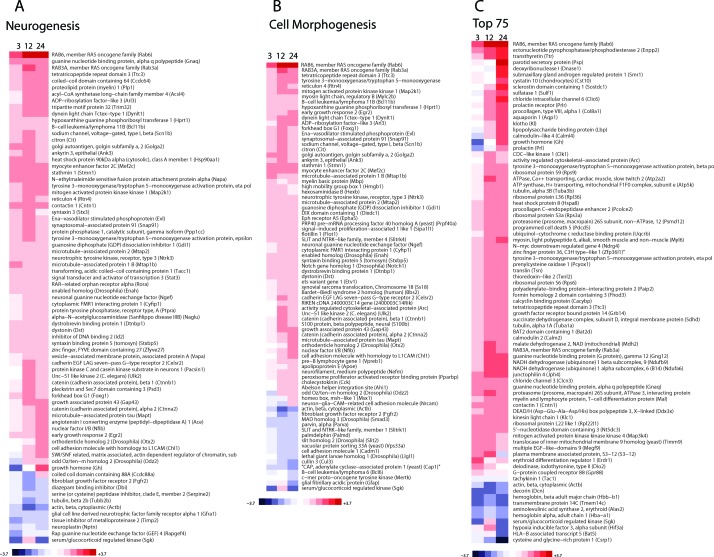
Hippocampal gene expression profiling of mRNAs in mice exposed to acute stress at 3 h, 12 h and 24 h The mice were sacrificed at 3, 12 and 24 h following restraint treatment as described in [20]. These mice therefore represented four discrete experimental groups: control (basal levels), 3 h acute stress, 12 h acute stress and 24 h acute stress (acute stressful levels). Hippocampal tissues were collected following the final stress exposure. The control samples were hippocampal tissues from mice not subjected to the restraint stress. Tissues were harvested under RNAse-free conditions to avoid RNA degradation as previously described [91]. Gene expression array profiling and analysis of microarray data were performed as in [20]. Fold changes were determined from log2 ratios between the probe signal in the control or exposed mouse. The log2 ratio was calculated for each probe set as the median of three biological replicates. Enriched transcripts from the GO terms ‘Neurogenesis’ **A.**, ‘Cell Morphology’ **B.** are shown. Additionally, transcripts were subsequently sorted by their importance in descending order of the sum-squared statistic (i.e., sum of squares of log2 ratios across the chronic time course) and the top 75 plotted **C.**. For the heat maps the range of colors is between −3.7-fold and +3.7-fold and preserves qualitative relationships among individual values. All fold changes outside of this range have been truncated to ± 3.7.

Neurogenesis, the process by which neurons are generated from neural stem cells and progenitor cells was among the most significantly enriched GO biological process terms (Bonferroni corrected *p*-value = 7.1E−09). We therefore explored the expression patterns of transcripts that play key roles in neurogenesis (Figure 2A). Many of the transcripts were upregulated by 3 h stressor exposure, such as components of the RAS oncogene family (*Rab6*, *Rab3a*), the guanine nucleotide binding protein alpha q polypeptide (*Gnaq*), contactin 1 (*Cntn1*), myocyte enhancer factor 2 C (*Mef2c*), stathmin 1 (*Stmn1*) and N−ethylmaleimide sensitive fusion protein attachment protein alpha (*Napa*). Actin beta (*Actb*), serum/glucocorticoid regulated kinase (*Sgk*) and growth hormone (*Gh*) were all down regulated 3 h after acute stress induction. Another significantly enriched GO biological process term was cell morphogenesis (Bonferroni corrected *p*-value = 1.1E-03) (Figure 2B) of which *Rab6*, *Rab3a*, *Mef2c*, *Stmn1*, *Actb*, *Sgk* and *Gh* are members.

## ACUTE STRESS INDUCES THE EXPRESSION OF GENES INVOLVED IN NEUROGENESIS, NEUROPROTECTION AND OXIDATIVE STRESS DEFENSE, AND DOWN-REGULATES GENES IMPLICATED IN NEURODEGENERATIVE DISEASES AND NEURONAL DYSFUNCTION

In addition to exploring GO enriched terms, the top ranked 75 probe sets were individually examined as to their behavior across the time course. A heat map was generated with these transcripts which show either strong down- or up-regulation in the acute group in comparison to control mice at the specific time points (Figure 2C). However, amongst them, we found that just a small portion of genes were down-regulated by acute stress.

### Enpp2, Ndufa6, Ndufb9 expression protects against oxidative stress

*Enpp2*, or autotaxin, is an enzyme with a lypophospholipase D activity which converts Lysophosphatidylcholine into Lypophosphatic Acid (*LPA*). Oxidative stress increases Ennp2 levels which, in turns, trigger an increment of LPA production in microglial cells [25, 26]. High LPA leads to inactivation of microglial cells reducing inflammation of the nervous system and protecting against oxidative stress-induced cellular damage. Consistent with this finding, the up-regulation of *Enpp2* in response to acute stress might be synonymous of cooperation between *Enpp2* and mRNAs involved in the respiratory chain, such as NADH dehydrogenase (ubiquinone) 1 beta subcomplex, 9 (*Ndufb9*) and NADH dehydrogenase (ubiquinone) 1 alpha subcomplex, 6 (B14) (*Ndufa6*), in coping with the oxidative stress. Indeed, both *Ndufb9* and *Ndufa6* were found highly expressed in the acute stress model under study (Figure 2C). Ndufb9 and Ndufa6 are subunits of the mitochondrial complex I. Mitochondrial complexes play important roles in energy metabolism and, therefore, a deficit in any one of them has an impact on specific body regions, such as the brain, with its high energy requirement. Impairment of any of the mitochondrial respiratory chain complexes including complex I and complex III, leads to a severe neurodegenerative disorder, Leigh syndrome, characterized by focal bilateral lesions in one or more areas of the central nervous system [27]. Moreover, mitochondrial dysfunction is a feature of AD brains, where an increase in mitochondrial membrane permeability and loss of membrane potential is associated with the release of cytochrome C and, in turns, apoptotic cell death [28]. Recurrence of restraint stress induces an increase in the lipid peroxide levels and a reduction of the total antioxidant reactivity in adult Wistar rats [29]. Whereas chronic stress can induce oxidative stress *via* a reduction in mitochondrial activity and is responsible for the deleterious effects observed in the hippocampus, acute stress enhances some components of the mitochondrial complexes and plays an important role in neuroprotection against oxidative stress [30].

### Rab6, Ttr, Gh, Prl, Jhp4, Calml4 expression improves neuroprotection, neurogenesis and cognitive functions

*Rab6*, the most strongly up-regulated transcript, 3 h following the acute stressor is a member of the RAS oncogene family. This small GTPase protein is located at the Golgi apparatus and regulates both anterograde and retrograde trafficking. Increased *Rab6* expression has been correlated with the unfolded protein response (UPR) of the endoplasmic reticulum (ER), an early event in the brain of AD patients aimed to restore ER homeostasis but damaging if not adequately regulated [31, 32]. A more recent study suggested that elevated levels of Rab6 are required for modulation of UPR to reduce the negative effects of prolonged ER stress [32]. Consequently, induction of *Rab6* expression by acute stress indicates the activation of a protective pathway against ER stress mediated toxicity which might be useful for stress management, thereby improving brain recovery.

Ttr is a homotetrameric protein involved primarily in the transport of thyroxin (T4) and vitamin A in cerebrospinal fluid (CSF) and plasma. According to published data on this protein, Ttr seems to play a dual role. On one hand, it is associated with neuronal protection and AD prevention but, on the other hand, up-regulation of *Ttr* correlates with a variety of neurodegenerative diseases [33, 34]. Mutated forms of Ttr have been associated with familial amyloid polyneuropathy (FAP) an autosomal dominant neurodegenerative disorder. Unfolded Ttr monomers self-aggregate generating deposition of amyloid fibrils that, in turn, leads to the apoptosis of neuronal cells [35]. It has also been demonstrated that modified Ttr, as major carrier of T4, can contribute to the development of multiple sclerosis. Indeed, high levels of Ttr characterized by specific oxidative modifications were detected in the CSF of multiple sclerosis patients with conversely reduced free T4 content [36]. Moreover, Ttr *in vitro* and *in vivo* experiments demonstrated that tissue-specific overexpression of *Ttr* ameliorates AD by inhibiting amyloid beta (Aβ) aggregation and thereby aiding detoxification [37]. Here we demonstrated using Illumina bead arrays, a time-dependent increase of *Ttr* in the hippocampus of mice that underwent an acute stressor. This finding was verified using Q-PCR analyses and another microarray platform from Applied Microarrays (data not shown), indicating a possible role for this protein in the cellular defense towards reactive oxygen species [38, 39]. In addition Ttr, due to its close relationship with thyroid hormones, is important not only during brain maturation but also in the adult vertebrate brain. Thyroid hormones are required for cell migration, dendrite and axon outgrowth, synapse formation, myelination and gliogenesis [40]. Hence, elevation of Ttr levels by acute stress affects thyroid hormone homeostasis leading to increased levels of T4 necessary for normal cytoskeletal assembly, stability and neuronal outgrowth [41].

Thyroid hormones interact with the growth hormone-insulin-like growth factor-1 axis (GH/IGF-I) to regulate development and growth. Dysfunction of GH/IGF-I axis or hypothyroidism causes hypothalamic or pituitary malformations, growth failure and psychosocial disorders [35]. In the adult brain, the GH/IGF-I axis affects cognition, biochemistry and neuroprotective effects [36]. In individuals with GH deficiency, the reduction in neural volume is significantly related to impaired cognitive function and motor skill abilities [38]. In this context, increased expression of *Gh* and *Ttr* mRNAs following one single restraint treatment corroborate the hypothesis that acute stress positively modulates neuron development and differentiation enhancing brain functions such as learning and memory [39].

Prolactin (*Prl*) and its receptor (*Prlr*) were down regulated initially at 3 h but up regulated at later time points. Prolactin is a cytokine as well as the primary lactogenic hormone, which functions as a neuromodulator and regulator of neuronal and glial plasticity in the brain [42]. Prl stimulates neurogenesis in the forebrain sub ventricular zone of pregnant female mice [43]. Moreover, chronically stressed mice treated with exogenous Prl show increased cell proliferation and survival of cells in the dentate gyrus compared to the control group [44]. Prl increases hippocampal precursor cells and its loss provokes learning and memory deficits, which are rescued by infusion of recombinant Prl into the hippocampus [45]. In addition, Prl together with Gh plays an important role in the regulation of the immune system. In fact, deficiencies in both cell-mediated and humoral immunological functions in hypophysectomized animals can be restored either by Prl or Gh [46]. Taken together, these findings suggest a pivotal role of Prl and Gh during neurogenesis, immune response and learning process implicating that increased levels of these hormones during acute stress have positive effects on the brain.

In this study, stressed mice showed elevated levels of Junctophilin 4 (*Jhp4*) mRNA relative to the control group. Jhp4 is involved in junctional membrane formation, which represents a subsurface cistern for the crosstalk between cell-surface and intracellular channels [47, 48]. Mutant mice lacking Jhp4 in hippocampal neurons have memory impairments and long-term potentiation (LTP) defects as well as hyperactivation of Ca^2+^/calmodulin-dependent protein kinase II. Thus, *Jhp4* up-regulation in the hippocampus may serve to preserve neuronal plasticity and integrity [49]. Furthermore, our microarray analysis showed that 12 h after stress treatment there was an increase of calmodulin like 4 (*Calml4*) mRNA levels. Calml4 belongs to the Ca^2+^/calmodulin family (calcium-binding protein) which modulates the Ca^2+^/calmodulin-dependent protein kinase II pathway. Aberrant regulation of this pathway has been associated with damaged learning, memory, synaptic plasticity and LTP [50, 51]. Indeed, as a calcium-binding protein, calmodulin acts as a signaling hub, mediating distribution of Ca^2+^ signals through a variety of effectors, modulating the different forms of synaptic plasticity [52]. A potential beneficial effect therefore might be generated by the synergistic increase of *Jhp4* and *Calml4* expression in response to acute stress aimed to strengthen brain activity. Interestingly, *Calml4* along with *Gh* and *Prl* were initially down regulated (3 h) and later strongly up-regulated (24 h).

### Sgk, Actb, Hbb-b1 and Hba-a1 impairment supports neurogenesis

Amongst the top 75 most strongly altered transcripts in response to the stressor, we found that just a small portion of genes were down-regulated by acute stress (Figure 2C). For us the most interesting ones, according to their functional role, were serum/glucocorticoid regulated kinase (*Sgk*), actin beta (*Actb*), hemoglobin, beta adult major chain (*Hbb−b1*) and hemoglobin alpha, adult chain 1 (*Hba−a1*).

Sgk is a kinase activated by insulin and growth factors *via* PI3K, PDK1 and mTORC2 pathways. It is involved in the modulation of enzymes and transcription factors such as GSK3-β, β-catenin and NF-kB. Moreover, it takes part in the regulation of transport, hormone release, neuroexcitability, inflammation, cell proliferation and apoptosis [40, 41, 53]. Inhibition of Sgk1 *via* a small molecule inhibitor, GSK650394, in a human hippocampal progenitor cell line suppressed the cortisol-induced reduction in neurogenesis [54]. This suggests that a reduction of *Sgk* mRNA upon acute stress might support neurogenesis.

The *Actb* gene encodes one of six different actin proteins. Actin exists as globular (G-actin) and filamentous (F-actin) forms and actin filaments can be either stable or dynamic. Development of the central nervous system requires changes in actin filaments which can have a critical role in the formation of new synapses and the maintenance of synaptic integrity [55]. Treatment with Aβ peptide induced actin stress fibers in the septal neuronal cell line SN1 and in primary cultured hippocampal neurons suggesting a role of actin cytoskeleton in the pathogenesis of AD Song [56].

The *Hbb−b1* and *Hba−a1* genes encode the beta polypeptide chain and the chain 1 subunits, respectively found in adult hemoglobin, which transports oxygen to various peripheral tissues. A study conducted on mice exposed to chronic and acute stressors showed that *Hbb−b1* and *Hba−a1* are over-expressed in response to chronic stress along with genes associated with the vascular system. This suggests that the hemoglobin genes contribute to brain damage and may potentially be used as chronic social stress biomarkers [57]. Overall, the reduction of *Sgk, Actb, Hbb−b1* and *Hba−a1* expression absolves a neuroprotective role. This finding was further explored by our GO analysis, which highlighted enrichment for neurogenesis related biological process terms (Figure 1A).

## ENRICHMENT OF PATHWAYS ASSOCIATED TO NEUROGENESIS AND COGNITIVE FUNCTIONS DURING ACUTE STRESS

Pathway analysis was carried out using the ToppGene Suite [58] and revealed significant enrichment of the following canonical pathways: ‘TNF-alpha/NF-kB signaling pathway’, ‘L13a-mediated translational silencing of ceruloplasmin expression’, ‘BDNF signaling pathway’, ‘regulation of Wnt-mediated beta catenin signaling and target gene transcription’, ‘parkin-ubiquitin proteasomal system pathway’, as well as ‘transmission across chemical synapses and glial cell differentiation’. In line with the GO analysis, the pathway analyses showed an enrichment of canonical pathways involved in neurogenesis and cognitive functions (Table 1).

The TNF-alpha/NF-kB signaling pathway has been shown to be crucial for neuronal generation, LTP and long term memory [59-61]. TNF-α induces *in vitro* proliferation of adult rat neural stem cells whereas its pharmacological blockade suppresses it [59]. Moreover, mice carrying a knockout of the subunit p50 NF-kB display alterations in late LTP and long-term memory [62, 63].

The Wnt/β-catenin signaling pathway has been associated with neurogenesis in adult hippocampal cells [64]. Overexpression of Wnt3 is enough to enhance neurogenesis from adult hippocampal stem/progenitor cells (AHPs) *in vitro* and *in vivo* [65]. In addition, several studies have shown that Wnt/β-catenin activation is protective against AD. Indeed, increased levels of GSK3β, which promotes β-catenin degradation, has been found in AD brains and correlates with neurodegeneration and deficiency in spatial learning [66-68]. In contrast, blockage of GSK3β, for instance by lithium, restores β-catenin levels reducing Aβ aggregates and astrogliosis explicating a protective effect against AD [69].

Noteworthy, the *brain-derived neurotrophic factor* (*BDNF*) gene is positively regulated by TNF-α/NF-kB and Wnt/β-catenin signaling pathways, thus it suggests a cooperation of these two pathways in the regulation of this growth factor [70-73]. Elevated levels of BDNF in the hippocampus promote spatial learning, memory, neurogenesis and neuroplasticity [74-77]. Conversely, diminished levels of BDNF are associated with psychiatric disorders such as, depression [78, 79].

Ceruloplasmin is involved in the regulation of the iron efflux, iron oxidation (Fe^2+^ to Fe^3+^) and stabilization of ferroportin membrane. L13a mediated translational silencing of ceruloplasmin expression seems to be beneficial for the brain, since ceruloplasmin up-regulation has been correlated with neurodegenerative diseases like schizophrenia and obsessive compulsive disorders [80, 81].

Parkin is a component of E3 ubiquitin ligase and belongs to the proteasomal system pathway [82]. Proteasome-mediated degradation has been shown to promote neuronal differentiation by down-regulation of a repressor of neuronal gene expression, REST [83]. Moreover, loss of functional Parkin leads to up-regulation of RTP801, which might be correlated with Parkinson's disease development [84]. Additionally, the enrichment in transmission across chemical synapses suggests that acute stress improves neuronal transmission also in agreement with the up-regulation of the neurotransmitter *Jhp4*.

**Table 1:** Pathway Analysis of Acute Stress Hippocampal Response

Category	ID	Name	Source	q-value (Bonferroni)	Hit Count in Query List	Hit Count in Genome
Pathway	83036	Ribosome	BioSystems: KEGG	3.49E-06	40	135
Pathway	198884	TNF-alpha/NF-kB Signaling Pathway	BioSystems: WikiPathways	6.15E-04	46	196
Pathway	105965	Translation	BioSystems: REACTOME	1.29E-03	47	207
Pathway	530764	Disease	BioSystems: REACTOME	1.09E-02	163	1088
Pathway	160955	L13a-mediated translational silencing of Ceruloplasmin expression	BioSystems: REACTOME	3.95E-02	36	161
Pathway	712093	BDNF signaling pathway	BioSystems: WikiPathways	5.19E-02	33	144
Pathway	169352	Regulation of Wnt-mediated beta catenin signaling and target gene transcription	BioSystems: Pathway Interaction Database	5.34E-02	22	79
Pathway	102279	Endocytosis	BioSystems: KEGG	6.01E-02	42	203
Pathway	700638	Parkin-Ubiquitin Proteasomal System pathway	BioSystems: WikiPathways	9.31E-02	21	76
Pathway	106516	Transmission across Chemical Synapses	BioSystems: REACTOME	9.39E-02	41	200
Pathway	698758	Glial Cell Differentiation	BioSystems: WikiPathways	9.43E-02	6	8
Pathway	187174	GABA synthesis, release, reuptake and degradation	BioSystems: REACTOME	1.70E-01	9	19
Pathway	198853	Cytoplasmic Ribosomal Proteins	BioSystems: WikiPathways	1.80E-01	28	121

## CONCLUSIONS AND FUTURE PERSPECTIVES

Stress is a normal physiological response of an organism to dangerous environmental stimuli. Acute stress provides many beneficial advantages whereas long-term or chronic stress has many well-documented negative effects [85-87]. As the majority of studies to date have focused on the impairment of the hippocampus by chronic stress mechanisms [18, 20, 88], we sought to examine a mouse model of acute stress, where animals exposed one time to a restraint stressor treatment were evaluated. The main goal of this study was to understand the positive effects of short-term stress on the hippocampus from a molecular point of view and uncover mRNA targets that were modulated by short-term stress. Our data suggest that acute stress stimulates the up-regulation of genes involved in neurogenesis and protection against oxidative stress which counteracts the oxidative DNA damage which is linked to many neurodegenerative diseases, including AD [89, 90]. In conclusion, acute stress appears to promote molecular machinery that acts to protect hippocampal neurons from the degenerative processes that can take place after a stressful event. Therefore, functional validation of acute stress targets could provide a new insight on cognitive disorders, mental illness and neurodegenerative diseases aimed to the development of more efficient therapies.
